# Supplementary figures and images for: The Effect of Genomic DNA Contamination on the Detection of Circulating Long Non-Coding RNAs: The Paradigm of MALAT1
Source: Diagnostics (Basel). 2021 Jun 25;11(7):1160. doi: 10.3390/diagnostics11071160 (PMC8305527; doi:10.3390/diagnostics11071160)

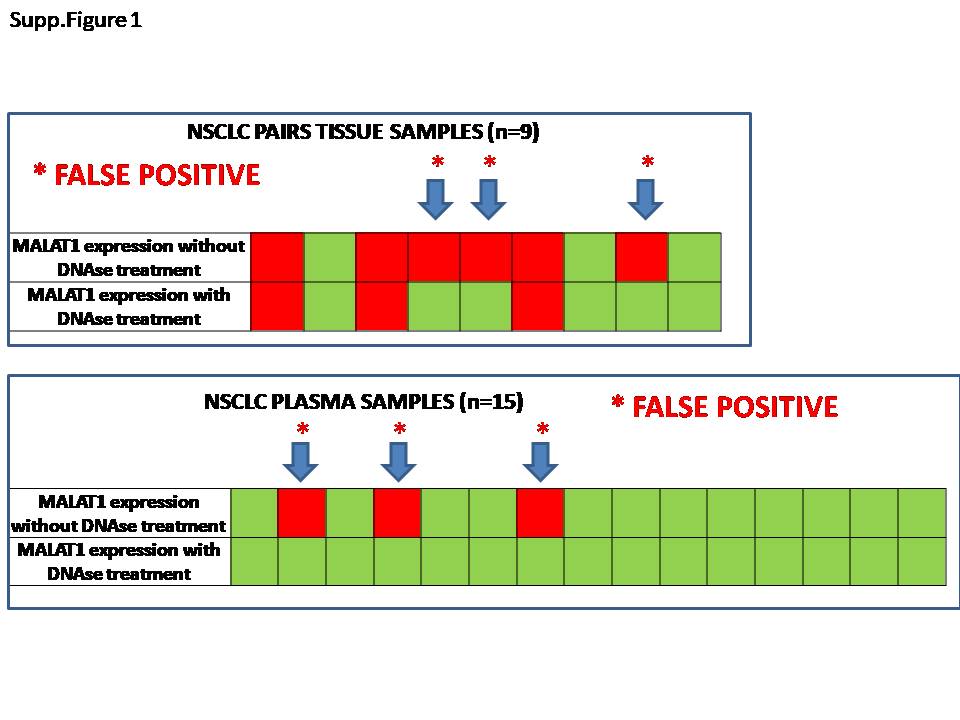

Supplement: Supplementary file 1 [file diagnostics-11-01160-s001.zip › diagnostics-1208534-supplementary.JPG]
